# P-423. High Burden of Intestinal Colonization with Carbapenem-Resistant Organisms (CRO) at two Greek Intensive Care Units

**DOI:** 10.1093/ofid/ofae631.624

**Published:** 2025-01-29

**Authors:** Olympia Zarkotou, Christina Louka, Georgios Alexandros Baziotis, Anna Roussou, Smaragdi Charami, Sophia Vourli, Panagiota Christina Georgiou, Styliani Louka, Elisavet Kousouli, Vasiliki Mamali, Polyxeni Karakosta, Spyros Pournaras

**Affiliations:** Tzaneio General Hospital of Piraeus, Piraeus, Attiki, Greece; Tzaneio General Hospital of Piraeus, Piraeus, Attiki, Greece; “Attikon” University General Hospital, Athens, Attiki, Greece; Tzaneio General Hospital of Piraeus, Piraeus, Attiki, Greece; Tzaneio General Hospital of Piraeus, Piraeus, Attiki, Greece; National and Kapodistrian University of Athens, Athens, Zakinthos, Greece; Attikon University General Hospital, Medical School, National and Kapodistrian University of Athens, Athens, Greece, Athens, Attiki, Greece; “Attikon” University General Hospital, Athens, Attiki, Greece; Tzaneio General Hospital of Piraeus, Athens, Greece., Piraeus, Attiki, Greece; Tzaneio General Hospital of Piraeus, Piraeus, Attiki, Greece; Attikon University General Hospital, Medical School, National and Kapodistrian University of Athens, Athens, Greece, Athens, Attiki, Greece; Attikon University General Hospital, Medical School, National and Kapodistrian University of Athens, Athens, Attiki, Greece

## Abstract

**Background:**

Screening for CRO intestinal colonization is part of the recommended interventions to limit their spread. We estimated the burden of carbapenem-resistant Enterobacterales (CRE), carbapenem-resistant *Acinetobacter baumannii* (CRAB) and carbapenem-resistant *Pseudomonas aeruginosa* (CRPA) intestinal colonization at two Greek ICUs: Attikon University Hospital (AUH) and Tzaneio General Hospital of Piraeus (TGHP).

**Figure:**
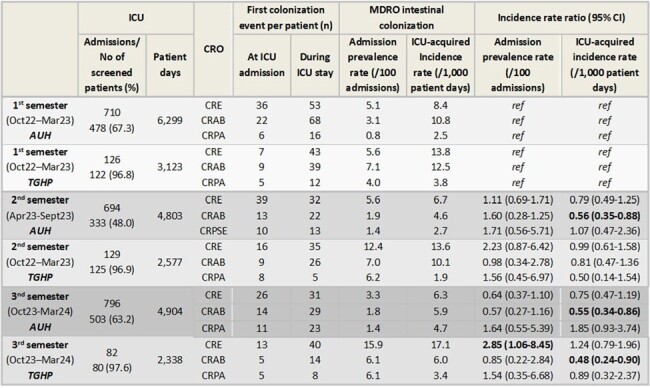

**Methods:**

From October 2022, all ICU patients were screened for intestinal colonization by CRO; patients admitted post-surgery on a daily basis were excluded. The first isolate per patient was recorded as a colonization event, categorized as positive either at admission (< 3 days from ICU admission) or during ICU stay ( >=3 days). Admission prevalence rates per 100 admissions (APR) and incidence rates of ICU-acquired intestinal colonization per 1,000 patient-days (IR) were calculated. Rates from the 1^st^, 2^nd^ and 3^rd^ study semester were compared and incidence rate ratios were determined.

**Figure:**
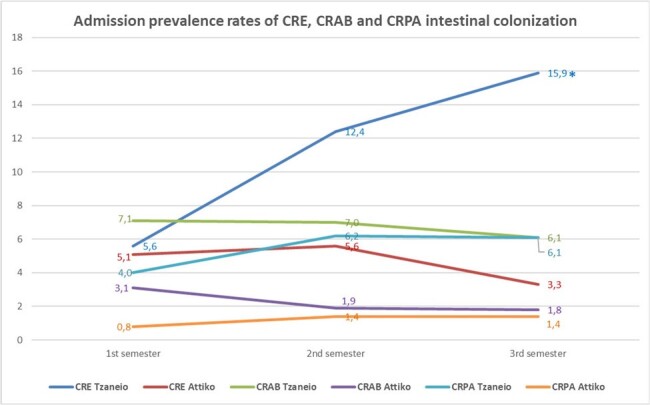

**Results:**

APR and IR of ICU-acquired CRE, CRAB, and CRPA intestinal colonization are presented in Table 1. APR for CRO colonization remained stable, during the study period, with the exception of CRE at TGHP, which exhibited a significant increase (p=0.02). APR were significantly higher at TGHP for all 3 study periods apart from CRE in the first study semester (figure 1). IR of ICU-acquired CRE colonization were also significantly higher at TGHP, in all 3 semesters, possibly due to the higher colonization pressure (figure 2). The IR of ICU-acquired CRE and CRPA colonization remained essentially unchanged over the study period at each hospital. However, a significant reduction in the incidence density of ICU-acquired CRAB colonization was observed (figure 2).

**Figure:**
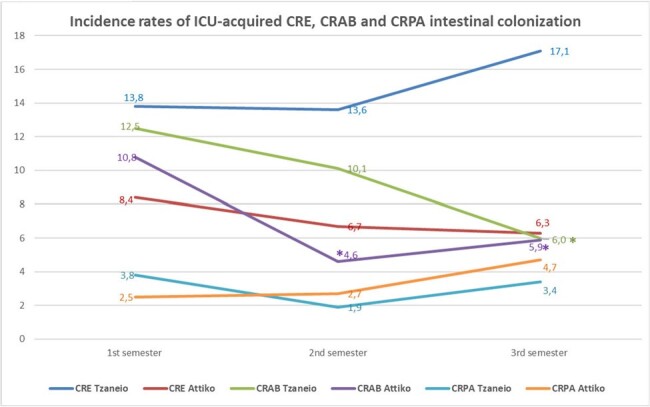

**Conclusion:**

Our study highlights the high burden of CRO colonization in Greek ICUs, which varies even across settings within the same region. CRE colonization presents the greater challenge, closely followed by CRAB, which however showed a significant reduction in ICU-acquired CRAB colonization during the last year. Active surveillance remains a crucial component of prevention bundles and can guide targeted prevention strategies.

**Disclosures:**

**All Authors**: No reported disclosures

